# Enhancing HIV Cognitive Abilities and Self-Management Through Information Technology–Assisted Interventions: Scoping Review

**DOI:** 10.2196/57363

**Published:** 2025-01-13

**Authors:** Hao Huang, MeiLian Xie, Zhen Yang, AiPing Wang

**Affiliations:** 1 School of Nursing Faculty of Health and Social Sciences Hong Kong Polytechnic University Hong Kong China (Hong Kong); 2 First Hospital of China Medical University Shenyang China; 3 Capital Medical University Beijing Ditan Hospital Beijing China

**Keywords:** digital media, self-management, HIV, AIDS, scoping review, technology-assisted interventions, information technology, behaviors, patient, electronic database, information systems, smartphone, app, SMS text messaging, effectiveness, mobile phone

## Abstract

**Background:**

HIV/AIDS remains a significant global challenge, and with the rapid advancement of technology, there has been an increasing number of interventions aimed at improving HIV/AIDS cognition and self-management behaviors among patients. However, there is still a lack of detailed literature integrating relevant evidence.

**Objective:**

This study aims to comprehensively review existing research on interventions using modern information methods to improve HIV/AIDS cognition and enhance self-management behaviors among patients. It systematically reports the theoretical frameworks and specific intervention strategies used in current research, providing a comprehensive overview of the development status of relevant studies. We aim to compile existing evidence through this scoping review to identify potential avenues for future research.

**Methods:**

We followed the scoping review framework proposed by the Joanna Briggs Institute for the synthesis and reporting of evidence. Relevant literature was searched using electronic databases, including PubMed, Web of Science, Embase, CINAHL, and Cochrane Library. The time frame for inclusion was from 2018 to December 1, 2023. Inclusion criteria were (1) interventions using modern information technology or new digital media, (2) studies focusing on improving HIV awareness or self-management behaviors among people living with HIV, (3) intervention studies or evaluations of intervention effects, and (4) studies published within the last five years. Two reviewers (HH and MX) independently assessed each study at both the title and abstract screening stage and the full-text review stage, resolving any disagreements through discussion.

**Results:**

A total of 55 studies that met the inclusion criteria were included. The Information-Motivation-Behavioral Skills model, Social Cognitive Theory, Health Belief Model, Theory of Planned Behavior, and Information Systems Research Framework are among the most commonly used theoretical frameworks. Modern information technology interventions are mainly constructed using smartphone apps, SMS text messaging, internet-based platforms, audiovisual materials, and digital health education platforms, with smartphone apps and SMS text messaging being the most widely used intervention media.

**Conclusions:**

Modern information technology is becoming an important tool for health interventions among people living with HIV/AIDS. However, future research should focus on integrating theoretical framework guidance with intervention design, further exploring the diversity of intervention implementations, the applicability of different technological methods, their long-term effects, and how to more effectively combine traditional intervention strategies to maximize intervention outcomes.

## Introduction

Currently, HIV/AIDS remains a severe and intricate global challenge. According to the 2023 report from the United Nations Program on HIV/AIDS [1], there are currently 39 million people living with HIV worldwide, with 29.8 million undergoing antiretroviral therapy (ART). In 2022, there were 1.3 million new HIV infections, and 630,000 individuals succumbed to AIDS-related illnesses. Thanks to collaborative efforts globally, the spread of HIV has slowed, and the annual death toll continues to decline [1,2]. Beyond the typical clinical symptoms of AIDS, patients often grapple with multidimensional challenges in their daily lives, including poor treatment adherence, exacerbated family conflicts, economic burdens due to high treatment costs, and mental health issues [3,4]. Research indicates that reinforcing self-management behaviors and enhancing disease awareness can lead to better treatment adherence, alleviate the burden of disease symptoms, and enable individuals to coexist more effectively with HIV [5,6]. Therefore, strengthening the self-management behaviors and disease awareness of people living with HIV becomes a crucial aspect of improving the quality of life for patients and reducing negative experiences associated with HIV.

Modern information technology rapidly and cost-effectively reduces the barriers to accessing disease-related information. It has been proven that leveraging modern technological means aids in expanding the dissemination of HIV prevention and treatment interventions [7]. Modern information technology, or new digital media, refers to user-controlled, shareable, and digital media [8]. Widely used new digital media, such as SMS text messages, websites, apps, and social media platforms, have significantly improved information sharing and communication. These technologies enable rapid, inexpensive, highly replicable, and widely covered interventions. Digital media achieve greater anonymity, repeatability, time flexibility, and customer sensitivity, meeting diverse learning needs [9]. They break down restrictions related to time and geography, maximizing the impact of information dissemination.

This study will comprehensively review evidence from the last 5 years regarding the use of modern information technology in enhancing HIV disease awareness and self-management. It underscores the enormous potential of contemporary technology in elevating self-management and disease awareness within the HIV community, guiding future research and practice.

## Methods

### Study Design

This scoping review uses the latest framework proposed by the Joanna Briggs Institute, encompassing the following stages: (1) formulation of research questions; (2) identification of relevant studies; (3) selection of studies; (4) charting the data; and (5) collating, summarizing, and reporting the results [10]. Reporting for this paper follows the PRISMA-ScR (Preferred Reporting Items for Systematic Reviews and Meta-Analyses Extension for Scoping Reviews) checklist, the preferred reporting item for scoping reviews [11]. All members of our research team have undergone systematic training in evidence-based research methodologies and are well-versed in conducting scoping reviews following established guidelines. Additionally, our team includes an expert in HIV research (MX), who has been available for consultation and reference throughout the study to ensure the quality and accuracy of our work. This scoping review has been registered on the Open Science Framework [12].

### Formulating Research Questions

This study aims to summarize existing evidence regarding enhancing HIV disease awareness and self-management behaviors among people living with HIV through modern information technology.

### Identifying Relevant Studies

We included studies that met the following criteria in this scoping review: (1) intervention involving modern information technology or new digital media, (2) research focusing on improving HIV disease awareness or self-management behaviors of people living with HIV, (3) intervention studies or studies evaluating intervention effects, and (4) studies published within the last five years. The exclusion criteria were (1) studies not reporting research outcomes, (2) gray literature, and (3) studies inaccessible for full-text retrieval.

We limited our literature search to studies published in 5 years to capture the most recent advancements in modern information technology interventions for improving disease awareness and self-management among people living with HIV/AIDS. This 5-year timeframe was chosen because the field is rapidly evolving, and recent studies are more likely to reflect current technologies and practices relevant to our research objectives [13].

### Study Selection

English-language literature was retrieved from electronic databases, including PubMed, Web of Science, Cochrane Library, Embase, and CINAHL. The literature search in this study focused on HIV, telemedicine, cognitive training, and self-management. For specific search strings, please refer to Multimedia Appendix 1. EndNote (Clarivate Analytics) software was used to manage the studies and eliminate duplicate publications. After removing duplicates, the literature underwent a title and abstract screening, followed by a full-text review to determine inclusion or exclusion. Both stages were performed independently by two reviewers (HH and MX), and no disagreements were reported.

To ensure consistency among reviewers, we randomly selected 25 titles and abstracts for an initial screening exercise. All reviewers independently screened these based on predefined inclusion criteria. Formal literature screening commenced once a 75% interrater agreement was achieved [14]. Subsequently, HH and MX independently screened the remaining titles and abstracts using the criteria and conducted full-text reviews to determine inclusion or exclusion. In cases of disagreement between the two reviewers, a third reviewer was consulted to discuss and reach a consensus [14]. Excluded studies are documented and reported, and the results and search process are presented in a flowchart following the PRISMA-ScR guidelines (Multimedia Appendix 2) [11].

### Data Extraction and Presentation

We conducted a comprehensive review of the full texts of studies meeting the inclusion criteria, focusing on how they enhanced disease awareness and self-management behaviors of patients with HIV through the application of theoretical frameworks and modern information technology interventions. Specifically, we extracted and reported on the theoretical frameworks and modern information technology used in each study.

Two researchers (HH and MX) independently extracted data and performed content analysis of the included studies. Any disagreements were resolved through discussion, with a third researcher (ZY) serving as an arbiter when necessary. Data were extracted and integrated using mind maps and Excel (Microsoft Corp) spreadsheets. The findings were then presented using a combination of text and tables.

We categorized and extracted the following information from each study: (1) basic study information, which includes authors, publication year, study design, sample size, and study location; (2) intervention methods, which includes details of the modern information technology interventions used in the studies; and (3) conceptual frameworks or models, which includes theoretical frameworks or models applied in the studies.

### Collating, Summarizing, and Reporting Results

We reported the results of the included studies by focusing on the following points: (1) the theoretical frameworks or models used in the included literature, and (2) intervention methods based on modern information technology adapted in the included literature.

## Results

### Study Selection

**Figure 1:**
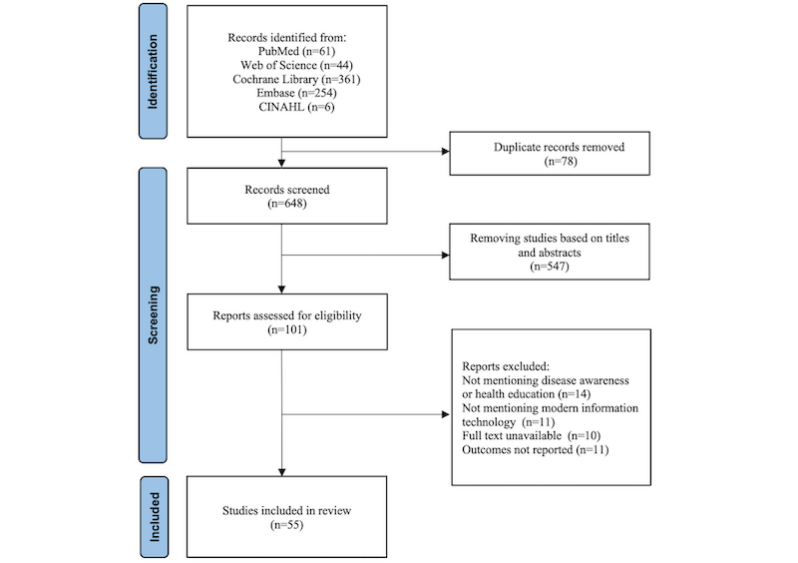
PRISMA-ScR (Preferred Reporting Items for Systematic Reviews and Meta-Analyses Extension for Scoping Reviews) flow diagram.

According to our inclusion criteria, the results of English database searches are as follows: 61 studies were retrieved from PubMed, 44 studies from Web of Science, 361 studies from Cochrane Library, 254 studies from Embase, and 6 studies from CINAHL. After removing duplicates, a total of 648 studies remained. After evaluating titles and abstracts, 101 English-language studies were retained, and after a thorough examination of the complete texts, 55 studies were finally included. The search results are presented as a PRISMA-ScR flowchart (Figure 1).

### Characteristics of the Studies

Among the 55 studies finally included, the studies ranged from a minimum sample size of 21 participants to a maximum of 2125 participants. Of these, 33 studies were randomized controlled trials, 10 studies were feasibility studies, 5 studies were observational studies, 4 studies were evaluative qualitative studies, and 3 studies were mixed methods studies. Most of the studies were conducted in the United States (n=28), followed by Kenya (n=4). Other countries include China (n=3), South Africa (n=3), Nigeria (n=3), Uganda (n=2), Thailand (n=2), and others. The general characteristics of the study are presented in Multimedia Appendix 3 [15-69].

### Related Theoretical Frameworks

#### Information-Motivation-Behavioral Skills Theory

Information-Motivation-Behavioral Skills (IMB) theory is the most commonly used theoretical framework for designing interventions, with 7 studies basing their interventions on this theory. The IMB theory posits that information, motivation, and behavioral skills interact, with individuals acquiring information to understand the necessity of healthy behavior [70]. This, in turn, motivates them to desire and guide the adoption of such behavior. Ultimately, behavioral skills are used to transform motivation into concrete actions.

Five studies [15-19] used mobile phones as a platform, operationalizing the IMB model to enhance HIV self-management knowledge and beliefs. They developed apps or tailored SMS text messages aimed at improving HIV self-management behaviors and increasing HIV disease awareness among patients with HIV. Additionally, two studies [20,21] facilitated changes in HIV awareness and self-management behaviors by stimulating motivation, providing relevant information, and enhancing behavioral skills among intervention participants. These changes were implemented through websites and videoconferences.

#### Social Cognitive Theory

Five studies used the Social Cognitive Theory to develop their interventions. Social Cognitive Theory [71] emphasizes the importance of social learning, suggesting that individuals can acquire new knowledge and skills by observing others and adjusting and internalizing this learning through cognitive regulation and self-efficacy. In line with this theory, existing studies propose methods to enhance self-efficacy, including peer modeling and social persuasion, and build interventions based on these principles. Weitzman et al [22] and Côté et al [23] developed web-based interventions to improve self-management skills. Kalichman et al [24] and Tran et al [25] focused on individual differences among intervention recipients, providing personalized interventions based on the Social Cognitive Theory to enhance medication adherence and self-management skills. Kinuthia et al [26], using the Social Cognitive Theory, crafted intervention messages, integrated treatment adherence with other priorities, and delivered them as SMS text messages to intervention recipients.

#### Health Belief Model

Three studies developed their interventions based on the Health Belief Model. Despite sharing the same name, the Health Belief Model discussed in this review encompasses studies that different scholars have proposed. First, Tran et al [25] applied the Health Belief Model proposed by Becker [72] in his study. Throughout the research period and subsequent meetings, Tran et al [25] measured perceived severity and self-efficacy for comparison and sent personalized messages as cues for action. The theory-driven design helped in understanding the connections between patient behaviors and identifying clustered behaviors such as alcohol consumption and unemployment. Second, Kinuthia et al [26] applied the Health Belief Model modified by Janz and Becker [73]. Kinuthia used this model to compose messages providing care support to pregnant and postpartum women infected with HIV. Additionally, the Health Belief Model applied by Ditre et al [27] was proposed by Champion and Skinner [74]. Based on this model, the scholars provided computer-generated personalized feedback to modify smoking and opioid misuse behaviors in people living with HIV.

#### Theory of Planned Behavior

Two studies used the Theory of Planned Behavior to construct their interventions. The Theory of Planned Behavior [75] posits that one can effectively predict whether an individual will adopt a specific behavior by measuring and analyzing behavioral intentions, attitudes, subjective norms, perceived behavioral control, and actual behavior. Ditre et al [27] integrated the Theory of Planned Behavior along with other theories to construct a comprehensive intervention, predicting and improving smoking and opioid misuse behaviors in patients with HIV. Jiao et al [28] used this theory to design an intervention aimed at improving ART adherence among men who have sex with men (MSM).

#### Information Systems Research Framework

Two studies used the Information Systems Research Framework (ISR) as their theoretical foundation. The ISR [76] advocates for iterative cycles in the design and evaluation process to provide a systematic scientific design approach. Beauchemin et al [29] developed and evaluated the effectiveness of app intervention for improving self-management behaviors in people living with HIV based on the ISR. Similarly, Schnall et al [30] used ISR to create a mobile app to enhance ART adherence among people living with HIV.

#### Other Theories

In addition to the 5 theories mentioned above, a few have been used by researchers in intervention studies. Ezegbe et al [31], based on Rational Emotive Behavior Therapy Theory [77], used audiovisual interventions to enhance HIV disease awareness among high school students. Nestadt et al [32], based on Social Action Theory [78], developed a cartoon-based program for comprehensive psychosocial responses and treatment adherence intervention for adolescents who are HIV positive. Schnall et al [33], using Social Learning Theory [79], developed a mobile app to examine its effectiveness in reducing sexual risk behaviors among young men attracted to the same sex. Cordova et al [34] conducted a narrative intervention based on the Ecodevelopmental and Empowerment Framework [80,81], aiming to change risky drug use and behaviors among adolescents. Middleton et al [35] and Carroll et al [36], guided by the Capability, Opportunity, Motivation, and Behavior Model [82], developed SMS text messages to increase intervention recipients’ knowledge of HIV prevention. Côté et al [23] implemented a web-based intervention guided by the McGill Nursing Model [83]. Sun et al [37], grounded in Cognitive Behavioral Theory [84], developed an app and tested its effectiveness in promoting HIV prevention among transgender women. Weitzman et al [22] designed web-based interventions based on the Modified AIDS Risk Reduction Model [85], examining their effectiveness for the target population. Chenneville et al [38], using the Cognitive Theory of Multimedia Learning [86] and Dual Coding Theory [87], designed modules to enhance health literacy in young MSM regarding HIV retention care. Cordoba et al [39], guided by the Information System Success [88] framework, evaluated participants’ experiences and satisfaction with a self-made app. In the psychological education segment of a comprehensive intervention, Ditre et al [27] primarily used the Transtheoretical Model [89] as guidance. Aladin et al [40], based on the Integrated, Design, Assess, and Share [90] theoretical framework, combined behavioral theories with design thinking and developed a mobile app as an intervention. Tran et al [25], based on the Integrated Theory of Behavior Change [91], developed a smartphone app to promote treatment adherence and self-efficacy among people living with HIV. To better illustrate the findings of this study, we have summarized the frequency of theoretical frameworks applied in the included studies. The results are visually represented in Table 1, providing a clear overview of the theoretical models and their respective utilization.

**Table 1:** Frequency of theoretical frameworks used in the included studies.

Theoretical framework	Frequency, n
Information-Motivation-Behavioral Skills Theory	7
Social Cognitive Theory	5
Health Belief Model	3
Theory of Planned Behavior	2
Information Systems Research Framework	2
Capability, Opportunity, Motivation, and Behavior Model	2
Rational Emotive Behavior Therapy Theory	1
Social Action Theory	1
Social Learning Theory	1
Ecodevelopmental and Empowerment Framework	1
McGill Nursing Model	1
Cognitive Behavioral Theory	1
The Modified AIDS Risk Reduction Model	1
Cognitive Theory of Multimedia Learning	1
Dual Coding Theory	1
Information System Success	1
Transtheoretical Model	1
Integrated, Design, Assess, and Share	1
Integrated Theory of Behavior Change	1

### Intervention Methods Based on Modern Information Technology

#### Smartphone Apps

Smartphones have become widely popular globally with their high portability and rich features. In this review, it was found that interventions based on smartphone apps are the most favored among researchers. Among the included studies, 25 used smartphone apps as an intervention method.

This category’s most popular intervention method involves providing self-management plans or tools to the intervention participants through smartphone apps, facilitating improvements in self-management behaviors. This is commonly observed in efforts to enhance compliance with medical advice, improve awareness of HIV, and ameliorate HIV-related symptoms [15,29,36,41-44,67,68]. Two studies integrated phone and SMS text message modules to build apps to enhance treatment adherence [45,46]. Communication-oriented mobile apps, such as WhatsApp (Meta), WeChat (Tencent), and Line (LY Corporation), were used to create group discussions for implementing interventions [17,47,48]. Finally, one study used the entertainment features of smartphones to develop an iPhone platform game to enhance the participant’s compliance [49].

Additionally, some studies have developed apps for targeted populations to implement interventions. For HIV testing behavior, sexual education, and treatment adherence among gay men, four studies have collectively developed three apps for interventions [33,39,50,51]. Two studies have developed apps targeting vulnerable adolescent groups, aiming to improve self-management behaviors and prevent disengagement from care [34,40]. In addressing health, one study developed an app to promote HIV prevention to meet the needs of transgender women [37].

#### SMS Text Message

Similar to interventions based on smartphone apps, using the SMS text message functionality of mobile phones for direct interventions has gained favor among many researchers. Sending SMS text messages to improve treatment adherence is the simplest and most direct method. Three studies have systematically sent timed or instant messages to intervention participants, covering medication reminders, peer education, and web-based group discussions, to enhance ART adherence among people living with HIV [16,28,52].

The included literature also takes into account specific populations. For instance, communication strategies in SMS text message content are tailored based on local cultural backgrounds for implementing prevention programs targeting 18- to 22-year-olds [53]. A combination of regular educational sessions and weekly health education SMS text messages are organized to enhance HIV prevention behaviors and reduce risky behaviors in African American youth [54]. Regularly sending SMS text messages for infant health education and promoting treatment adherence is implemented to provide care support for pregnant women and postpartum women living with HIV [26]. Planned SMS text messaging campaigns are carried out to raise HIV awareness among construction workers and encourage HIV testing [35].

#### Web-Based and Digital Health Education Platforms

In internet-based interventions, most researchers choose to implement intervention programs by constructing websites. Building websites allows for the rapid dissemination of health education. Three studies used websites to create knowledge repositories for providing safety education. These websites comprehensively covered various topics, offering comprehensive and personalized health education to intervention participants through web-based interactions, sexual health information delivery, and modularized intervention methods. This approach aimed to promote HIV prevention and increase awareness of sexual health [55-57]. Using website platforms, two studies relied on teams with medical backgrounds to provide diverse learning methods. They combined social support and emphasized personalized web-based services to facilitate the effective transmission of health information [22,58]. Interventions providing counseling services through remote videoconferences typically use internet platforms to offer web-based video meetings to intervention participants. This involves hosting regular videoconferences and encouraging participant involvement through email, phone calls, and other means to enhance medical adherence and disease awareness among people living with HIV [21,23,59].

Furthermore, there are different forms of internet-based intervention programs. For example, one study created private discussion groups on social media platforms to enhance HIV-related knowledge and treatment literacy, thereby promoting the continuity of HIV care [60]. Other studies constructed internet-based electronic health education programs to strengthen health literacy among people living with HIV in HIV care through digital content [38], developed intervention programs using publicly available internet resources [20], and constructed HIV prevention interventions through video blogs that feature storytelling and digital gaming [61].

Finally, a minority of studies have implemented interventions by constructing digital health education platforms. These studies use computer-based personalized approaches, providing tailored health information and training videos through digital platforms to enhance treatment adherence and HIV-related knowledge and improve health care behaviors among intervention participants [27,62,63].

#### Performing and Multimedia Interventions

The commonality of audio and video interventions is the use of virtualized digital content to create various intervention plans tailored to different populations and needs. These interventions are delivered in audio and video, emphasizing the dissemination of disease-related knowledge and fostering connections between individuals and communities. Ultimately, they aim to enhance the understanding of intervention participants, boost self-efficacy among people living with HIV, and improve preventive behaviors.

For the adolescent population, considering their capacity for acceptance and understanding, researchers have developed a series of cartoon tutorials to strengthen parent-child communication, problem-solving, and negotiation skills. Participants pair with caregivers to attend the courses, enhancing internal family connections through learning and discussions [32]. For children, researchers have created captivating characters and carefully curated story content. With the assistance of qualified therapists, they deliver digital interventions to children using audio and video formats, achieving intervention goals while providing entertainment [64].

**Figure 2:**
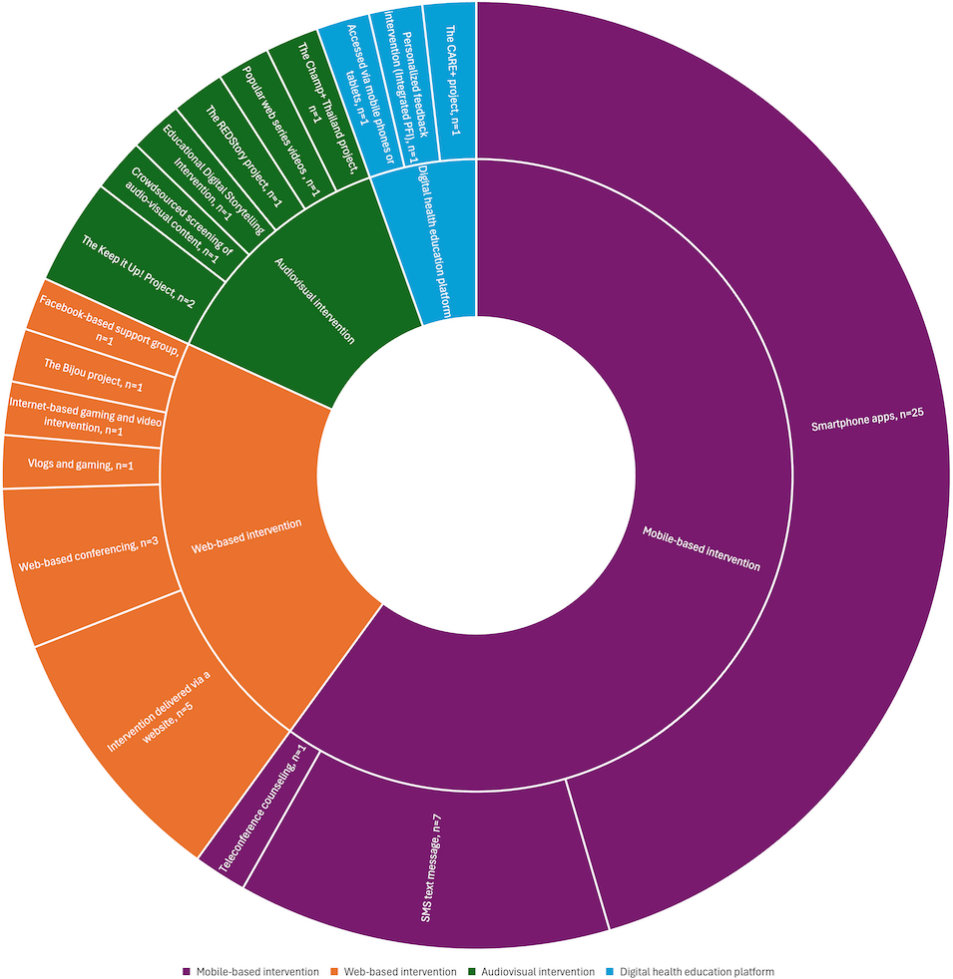
Visualization of interventions included in the studies by type. PFI: personalized feedback intervention.

For specific populations, such as the gay and bisexual community, researchers have developed popular web series or short videos with a theme focused on gay, bisexual, and other MSM individuals. These videos capture the curiosity of the target audience while promoting HIV and other sexually transmitted infection testing behaviors [65,66,69]. Finally, some studies combine various communication channels, such as digital games, street interviews, and therapist-guided group psychological discussions, to enhance the effectiveness of performing multimedia interventions, providing participants with knowledge about preventing HIV/AIDS and sexually transmitted infections [18,19,31]. We have visualized the interventions included in the studies through Figure 2 to present the findings of this section more intuitively.

## Discussion

### Principal Findings

This scoping review reveals that most of the included studies have used one or more theoretical frameworks in the development of their interventions. Currently, the application of modern information technology primarily involves intervention construction, development of smartphone apps, feasibility studies, and analysis to intervene with the relevant population, yielding generally positive feedback. Thanks to the rapid advancement of modern technology, there has been an increasing emergence of modern information technologies, which better improve or innovate medical intervention measures and hold significant potential for the future development of medical technology. Modern information technology (or new media technology) plays a crucial role in HIV/AIDS research. This study explores the potential of information technology in enhancing HIV/AIDS awareness from multiple dimensions and systematically summarizes the theoretical frameworks and specific implementation methods of relevant studies. Through this research, we not only summarize the application cognition of modern information technology in health intervention but also provide new ideas and methods to improve HIV/AIDS awareness among older people, which is meaningful for enhancing HIV/AIDS awareness and inspiring future related research.

As early as 2000, scholars reported that theory-driven intervention measures have been proven to be more effective than non–theory-driven ones [92]. However, among the 55 studies included in this study, only 45% (n=25) of the intervention schemes were based on theory-driven approaches, with the remaining studies either not reporting the research framework used or not using any theoretical framework. Among these 25 studies, the most commonly used theory was the IMB theory [70]. In health education interventions based on the IMB theory, accurate information about HIV/AIDS is first provided, emphasizing personalized interventions to increase individual awareness levels. Subsequently, by highlighting the serious threat of HIV/AIDS to individual health, individuals’ positive motivations for prevention and treatment are stimulated, encouraging proactive behavior change. Finally, by providing practical behavioral skills training, individuals are helped to overcome barriers to implementing healthy behaviors, such as correctly using condoms and undergoing regular HIV testing, to achieve the intervention’s objectives. The widespread application of the IMB theory in HIV/AIDS health education reflects its effectiveness in integrating information, motivation, and behavioral skills, providing theoretical support for designing targeted and effective health interventions. Additionally, some studies have integrated multiple theoretical frameworks as comprehensively as possible to construct intervention schemes [22,23,26,38,43]. Therefore, we encourage future research to use theory-driven approaches as much as possible to enhance the quality of interventions and improve intervention outcomes.

We found that the majority of studies used mobile phones as a platform for cognitive interventions, demonstrating diversity and innovation in HIV/AIDS cognitive interventions. From the results of this study, it is evident that the intelligent apps developed by various studies transcend the traditional role of communication tools in cognitive interventions. Leveraging the high portability and ubiquity of smartphones, these interventions offer convenient and personalized ways to reach the target population. By harnessing the mature functionalities of smartphones, such as integrating phone calls, SMS text messaging modules, and chat apps, these interventions provide communication pathways and methods rooted in traditional communication for intervention purposes [17,45-48]. The customization of apps tailored to specific populations, such as MSM [33,39,50,51], vulnerable adolescents [40], and transgender women [37], highlights the flexibility and adaptability of smartphone apps in meeting the diverse health needs of different groups.

### Future Directions

Despite the promising future and wide-ranging applicability of smartphone app interventions, as evident from the studies included in this scoping review, only one study [45] mentions the relevance to the population of older people. Older people may face unique challenges and needs related to HIV/AIDS, such as unfamiliarity or discomfort with digital technology and preferences for accessing health information through specific channels. Therefore, developing specialized smartphone app interventions tailored to the population of older people is essential. These interventions should address the unique needs and preferences of older adults by incorporating user-friendly features that align with their specific contexts and lifestyles. For example, simplified navigation systems; larger font sizes; and real-time, one-on-one, web-based support are highly recommended. Additionally, for older people who may face challenges with typing or using smartphones, voice-guided navigation could be an especially effective solution. Such tailored interventions and innovations are crucial for enhancing accessibility and usability, ultimately improving the older people population’s engagement with health information and interventions.

Through this review, it is evident that a significant portion of studies prefer using SMS text messaging for interventions, with only one study [16] emphasizing the importance of protecting user privacy. SMS text messaging interventions involve the collection, storage, and use of users’ personal information. Therefore, safeguarding user privacy is crucial, especially when sensitive health information is involved. The lack of adequate protection for user privacy may lead to distrust and resistance among users, impacting their acceptance and engagement with the intervention.

### Strengths and Limitations

To the best of our knowledge, this scoping review is the first to comprehensively summarize the theoretical frameworks used in interventions using modern information technologies to enhance HIV/AIDS-related cognition and self-management behaviors. Adhering to the Joanna Briggs Institute framework provides a structured and standardized approach to the scoping review process, enhancing the reliability and reproducibility of the findings. Additionally, it provides an updated integration of recent intervention studies leveraging contemporary digital tools, reflecting the rapid advancements in information technology. By systematically consolidating existing intervention strategies, this review offers diverse possibilities for future research and helps prevent the redundancy of developing similar intervention schemes.

This scoping review has several limitations. First, we excluded gray literature, such as conference abstracts and dissertations, from our inclusion and exclusion criteria, which may have restricted the literature retrieved during the search. Second, all the studies included in the review were in English, potentially overlooking high-quality studies written in other languages. Furthermore, some studies using modern information technologies may have used different keywords, which could have resulted in the omission of relevant research from our review. Finally, as a scoping review, this study does not assess the effectiveness of the interventions, which should be addressed in future systematic reviews or meta-analyses.

### Conclusions

This study found that modern information technology has been widely used in designing intervention programs to improve cognitive function and self-management behaviors among people living with HIV/AIDS. These interventions primarily use mobile phones as carriers and the internet as the medium for dissemination. Such technological approaches enable personalized, convenient, and real-time interventions, expanding the methods and avenues of traditional interventions. However, we also observed that most of the included studies have not fully used existing theories or conceptual frameworks in constructing their interventions. The lack of support from theoretical frameworks may lead to research failing to achieve the established goals as originally designed, thereby slowing the problem-solving process and potentially affecting the final effectiveness.

## Appendix

Multimedia Appendix 1 Specific search strings.

Multimedia Appendix 2 PRISMA-ScR (Preferred Reporting Items for Systematic Reviews and Meta-Analyses Extension for Scoping Reviews) checklist.

Multimedia Appendix 3 The general information of included literature.

## Abbreviations

ART: antiretroviral therapy
IMB: Information-Motivation-Behavioral Skills
ISR: Information Systems Research Framework
MSM: men who have sex with men
PRISMA-ScR: Preferred Reporting Items for Systematic Reviews and Meta-Analyses Extension for Scoping Reviews

## Data Availability

The datasets generated during or analyzed during this study are available from the corresponding author upon reasonable request.

## References

[1] (2023). The path that ends AIDS: UNAIDS global AIDS update 2023. UNAIDS.

[2] UNAIDS global AIDS update 2022 reveals millions of lives are “in danger”. U.S. Embassy in Mozambique.

[3] Wanjala SW, Nyongesa MK, Mapenzi R, Luchters S, Abubakar A (2023). A qualitative inquiry of experiences of HIV-related stigma and its effects among people living with HIV on treatment in rural Kilifi, Kenya. Front Public Health.

[4] Nyongesa MK, Nasambu C, Mapenzi R, Koot HM, Cuijpers P, Newton CRJC, Abubakar A (2022). Psychosocial and mental health challenges faced by emerging adults living with HIV and support systems aiding their positive coping: a qualitative study from the Kenyan coast. BMC Public Health.

[5] King E, Kinvig K, Steif J, Qiu AQ, Maan EJ, Albert AY, Pick N, Alimenti A, Kestler MH, Money DM, Lester RT, Murray MCM (2017). Mobile text messaging to improve medication adherence and viral load in a vulnerable Canadian population living with human immunodeficiency virus: a repeated measures study. J Med Internet Res.

[6] Moucheraud C, Stern AF, Ismail A, Nsubuga-Nyombi T, Ngonyani MM, Mvungi J, Ssensamba J (2020). Can self-management improve HIV treatment engagement, adherence, and retention? A mixed methods evaluation in Tanzania and Uganda. AIDS Behav.

[7] Young SD, Cumberland WG, Nianogo R, Menacho LA, Galea JT, Coates T (2015). The HOPE social media intervention for global HIV prevention in Peru: a cluster randomised controlled trial. Lancet HIV.

[8] Guse K, Levine D, Martins S, Lira A, Gaarde J, Westmorland W, Gilliam M (2012). Interventions using new digital media to improve adolescent sexual health: a systematic review. J Adolesc Health.

[9] Marsch LA, Grabinski MJ, Bickel WK, Desrosiers A, Guarino H, Muehlbach B, Solhkhah R, Taufique S, Acosta M (2011). Computer-assisted HIV prevention for youth with substance use disorders. Subst Use Misuse.

[10] Peters MDJ, Marnie C, Tricco AC, Pollock D, Munn Z, Alexander L, McInerney P, Godfrey CM, Khalil H (2020). Updated methodological guidance for the conduct of scoping reviews. JBI Evid Synth.

[11] Tricco AC, Lillie E, Zarin W, O'Brien KK, Colquhoun H, Levac D, Moher D, Peters MDJ, Horsley T, Weeks L, Hempel S, Akl EA, Chang C, McGowan J, Stewart L, Hartling L, Aldcroft A, Wilson MG, Garritty C, Lewin S, Godfrey CM, Macdonald MT, Langlois EV, Soares-Weiser K, Moriarty J, Clifford T, Tunçalp Ö, Straus SE (2018). PRISMA extension for scoping reviews (PRISMA-ScR): checklist and explanation. Ann Intern Med.

[12] Huang H, Yang Z, Xie M, Wang A (2023). Information technology-assisted intervention for HIV cognitive enhancement and self-management: a scoping review. Open Science Framework.

[13] Levac D, Colquhoun H, O'Brien KK (2010). Scoping studies: advancing the methodology. Implement Sci.

[14] Peters MDJ, Godfrey C, McInerney P, Munn Z, Tricco AC, Khalil H, Aromataris E, Munn Z (2020). Chapter 11: scoping reviews (2020 version). JBI Manual for Evidence Synthesis.

[15] Shim MS, Kim S, Choi M, Choi JY, Park CG, Kim GS (2022). Developing an app-based self-management program for people living with HIV: a randomized controlled pilot study during the COVID-19 pandemic. Sci Rep.

[16] Aunon FM, Wanje G, Richardson BA, Masese L, Odeny TA, Kinuthia J, Mandaliya K, Jaoko W, Simoni JM, McClelland RS (2023). Randomized controlled trial of a theory-informed mHealth intervention to support ART adherence and viral suppression among women with HIV in Mombasa, Kenya: preliminary efficacy and participant-level feasibility and acceptability. BMC Public Health.

[17] Fan X, Ning K, Liu C, Zhong H, Lau JTF, Hao C, Hao Y, Li J, Li L, Gu J (2023). Uptake of an app-based case management service for HIV-positive men who have sex with men in China: process evaluation study. J Med Internet Res.

[18] Madkins K, Moskowitz DA, Moran K, Dellucci TV, Mustanski B (2019). Measuring acceptability and engagement of the keep it up! internet-based HIV prevention randomized controlled trial for young men who have sex with men. AIDS Educ Prev.

[19] Mustanski B, Parsons JT, Sullivan PS, Madkins K, Rosenberg E, Swann G (2018). Biomedical and behavioral outcomes of keep it up!: an eHealth HIV prevention program RCT. Am J Prev Med.

[20] Whiteley LB, Brown LK, Curtis V, Ryoo HJ, Beausoleil N (2018). Publicly available internet content as a HIV/STI prevention intervention for urban youth. J Prim Prev.

[21] Navarra AMD, Rosenberg MG, Gormley M, Bakken S, Fletcher J, Whittemore R, Gwadz M, Cleland C, Melkus GD (2023). Feasibility and acceptability of the adherence connection counseling, education, and support (ACCESS) proof of concept: a peer-led, mobile health (mHealth) Cognitive Behavioral Antiretroviral Therapy (ART) Adherence Intervention for HIV-Infected (HIV+) Adolescents and Young Adults (AYA). AIDS Behav.

[22] Weitzman PF, Zhou Y, Kogelman L, Mack S, Sharir JY, Vicente SR, Levkoff SE (2020). A web-based HIV/STD prevention intervention for divorced or separated older women. Gerontologist.

[23] Côté J, Rouleau G, Ramirez-Garcia MP, Auger P, Thomas R, Leblanc J (2020). Effectiveness of a web-based intervention to support medication adherence among people living with HIV: web-based randomized controlled trial. JMIR Public Health Surveill.

[24] Kalichman SC, Cherry C, Kalichman MO, Eaton LA, Kohler JJ, Montero C, Schinazi RF (2018). Mobile health intervention to reduce HIV transmission: a randomized trial of behaviorally enhanced HIV treatment as prevention (B-TasP). J Acquir Immune Defic Syndr.

[25] Tran BX, Bui TM, Do AL, Boyer L, Auquier P, Nguyen LH, Nguyen AHT, Ngo TV, Latkin CA, Zhang MWB, Ho CSH, Ho RCM (2023). Efficacy of a mobile phone-based intervention on health behaviors and HIV/AIDS treatment management: randomized controlled trial. J Med Internet Res.

[26] Kinuthia J, Ronen K, Unger JA, Jiang W, Matemo D, Perrier T, Osborn L, Chohan BH, Drake AL, Richardson BA, John-Stewart G (2021). SMS messaging to improve retention and viral suppression in prevention of mother-to-child HIV transmission (PMTCT) programs in Kenya: a 3-arm randomized clinical trial. PLoS Med.

[27] Ditre JW, LaRowe LR, Vanable PA, de Vita MJ, Zvolensky MJ (2019). Computer-based personalized feedback intervention for cigarette smoking and prescription analgesic misuse among persons living with HIV (PLWH). Behav Res Ther.

[28] Jiao K, Wang C, Liao M, Ma J, Kang D, Tang W, Tucker JD, Ma W (2022). A differentiated digital intervention to improve antiretroviral therapy adherence among men who have sex with men living with HIV in China: a randomized controlled trial. BMC Med.

[29] Beauchemin M, Gradilla M, Baik D, Cho H, Schnall R (2019). A multi-step usability evaluation of a self-management app to support medication adherence in persons living with HIV. Int J Med Inform.

[30] Schnall R, Sanabria G, Jia H, Cho H, Bushover B, Reynolds NR, Gradilla M, Mohr DC, Ganzhorn S, Olender S (2023). Efficacy of an mHealth self-management intervention for persons living with HIV: the wiseApp randomized clinical trial. J Am Med Inform Assoc.

[31] Ezegbe B, Eseadi C, Ede MO, Igbo JN, Aneke A, Mezieobi D, Ugwu GC, Ugwoezuonu AU, Elizabeth E, Ede KR, Ede AO, Ifelunni CO, Amoke C, Eneogu ND, Effanga OA (2018). Efficacy of rational emotive digital storytelling intervention on knowledge and risk perception of HIV/AIDS among schoolchildren in Nigeria. Medicine (Baltimore).

[32] Nestadt DF, Saisaengjan C, McKay MM, Bunupuradah T, Pardo G, Lakhonpon S, Gopalan P, Leu C, Petdachai W, Kosalaraksa P, Srirompotong U, Ananworanich J, Mellins CA (2019). CHAMP+ Thailand: pilot randomized control trial of a family-based psychosocial intervention for perinatally HIV-infected early adolescents. AIDS Patient Care STDS.

[33] Schnall R, Kuhns LM, Pearson C, Batey DS, Bruce J, Hidalgo MA, Hirshfield S, Janulis P, Jia H, Radix A, Belkind U, Rodriguez RG, Garofalo R (2022). Efficacy of MyPEEPS mobile, an HIV prevention intervention using mobile technology, on reducing sexual risk among same-sex attracted adolescent males: a randomized clinical trial. JAMA Netw Open.

[34] Cordova D, Munoz-Velazquez J, Lua FM, Fessler K, Warner S, Delva J, Adelman N, Fernandez A, Bauermeister J (2020). Pilot study of a multilevel mobile health app for substance use, sexual risk behaviors, and testing for sexually transmitted infections and HIV among youth: randomized controlled trial. JMIR Mhealth Uhealth.

[35] Middleton M, Somerset S, Evans C, Blake H (2020). Test@Work texts: mobile phone messaging to increase awareness of HIV and HIV testing in UK construction employees during the COVID-19 pandemic. Int J Environ Res Public Health.

[36] Carroll JK, Tobin JN, Luque A, Farah S, Sanders M, Cassells A, Fine SM, Cross W, Boyd M, Holder T, Thomas M, Overa CC, Fiscella K (2019). "Get Ready and Empowered About Treatment" (GREAT) study: a pragmatic randomized controlled trial of activation in persons living with HIV. J Gen Intern Med.

[37] Sun CJ, Anderson KM, Kuhn T, Mayer L, Klein CH (2020). A sexual health promotion app for transgender women (Trans Women Connected): development and usability study. JMIR Mhealth Uhealth.

[38] Chenneville T, Drake H, Gabbidon K, Rodriguez C, Hightow-Weidman L (2021). Bijou: engaging young MSM in HIV care using a mobile health strategy. J Int Assoc Provid AIDS Care.

[39] Cordoba E, Idnay B, Garofalo R, Kuhns LM, Pearson C, Bruce J, Batey DS, Radix A, Belkind U, Hidalgo MA, Hirshfield S, Rodriguez RG, Schnall R (2021). Examining the information systems success (ISS) of a mobile sexual health app (MyPEEPS Mobile) from the perspective of very young men who have sex with men (YMSM). Int J Med Inform.

[40] Aladin B, Thompson M, Addison D, Havens J, McGowan J, Nash D, Smith C (2023). The YGetIt? Program: a mobile application, PEEP, and digital comic intervention to improve HIV care outcomes for young adults. Health Promot Pract.

[41] Safdari R, SeyedAlinaghi S, Mohammadzadeh N, Noori T, Rahmati P, Qaderi K, Voltarelli F, Mehraeen E (2021). Developing Aysoo: a mobile-based self-management application for people living with HIV. HIV AIDS Rev.

[42] Clouse K, Noholoza S, Madwayi S, Mrubata M, Camlin CS, Myer L, Phillips TK (2023). The implementation of a GPS-based location-tracking smartphone app in South Africa to improve engagement in HIV care: randomized controlled trial. JMIR Mhealth Uhealth.

[43] Cho H, Flynn G, Saylor M, Gradilla M, Schnall R (2019). Use of the FITT framework to understand patients' experiences using a real-time medication monitoring pill bottle linked to a mobile-based HIV self-management app: a qualitative study. Int J Med Inform.

[44] Barroso J, Madisetti M, Mueller M (2020). A feasibility study to develop and test a cognitive behavioral stress management mobile health application for HIV-related fatigue. J Pain Symptom Manage.

[45] Morano JP, Clauson K, Zhou Z, Escobar-Viera CG, Lieb S, Chen IK, Kirk D, Carter WM, Ruppal M, Cook RL (2019). Attitudes, beliefs, and willingness toward the use of mHealth tools for medication adherence in the florida mHealth adherence project for people living with HIV (FL-mAPP): pilot questionnaire study. JMIR Mhealth Uhealth.

[46] Twimukye A, Naggirinya AB, Parkes-Ratanshi R, Kasirye R, Kiragga A, Castelnuovo B, Wasswa J, Nabaggala MS, Katabira E, Lamorde M, King RL (2021). Acceptability of a mobile phone support tool (Call for Life Uganda) for promoting adherence to antiretroviral therapy among young adults in a randomized controlled trial: exploratory qualitative study. JMIR Mhealth Uhealth.

[47] Bergam S, Sibaya T, Ndlela N, Kuzwayo M, Fomo M, Goldstein MH, Marconi VC, Haberer JE, Archary M, Zanoni BC (2022). "I am not shy anymore": a qualitative study of the role of an interactive mHealth intervention on sexual health knowledge, attitudes, and behaviors of South African adolescents with perinatal HIV. Reprod Health.

[48] Anand T, Nitpolprasert C, Jantarapakde J, Meksena R, Phomthong S, Phoseeta P, Phanuphak P, Phanuphak N (2020). Implementation and impact of a technology-based HIV risk-reduction intervention among Thai men who have sex with men using "Vialogues": a randomized controlled trial. AIDS Care.

[49] Whiteley L, Brown LK, Mena L, Craker L, Arnold T (2018). Enhancing health among youth living with HIV using an iPhone game. AIDS Care.

[50] Hightow-Weidman L, Muessig KE, Egger JR, Vecchio A, Platt A (2021). Epic allies: a gamified mobile app to improve engagement in HIV care and antiretroviral adherence among young men who have sex with men. AIDS Behav.

[51] Horvath KJ, Lammert S, Danh T, Mitchell JW (2020). The feasibility, acceptability and preliminary impact of mobile application to increase repeat HIV testing among sexual minority men. AIDS Behav.

[52] Mao L, Buchanan A, Wong HTH, Persson A (2018). Beyond mere pill taking: SMS reminders for HIV treatment adherence delivered to mobile phones of clients in a community support network in Australia. Health Soc Care Community.

[53] Ybarra ML, Agaba E, Nyemara N (2021). A pilot RCT evaluating InThistoGether, an mHealth HIV prevention program for Ugandan youth. AIDS Behav.

[54] Mayo-Wilson LJ, Coleman J, Timbo F, Latkin C, Brown ERT, Butler AI, Conserve DF, Glass NE (2020). Acceptability of a feasibility randomized clinical trial of a microenterprise intervention to reduce sexual risk behaviors and increase employment and HIV preventive practices (EMERGE) in young adults: a mixed methods assessment. BMC Public Health.

[55] Ivanova O, Wambua S, Mwaisaka J, Bossier T, Thiongo M, Michielsen K, Gichangi P (2019). Evaluation of the ELIMIKA pilot project: improving ART adherence among HIV positive youth using an eHealth intervention in Mombasa, Kenya. Afr J Reprod Health.

[56] Hightow-Weidman LB, LeGrand S, Muessig KE, Simmons RA, Soni K, Choi SK, Kirschke-Schwartz H, Egger JR (2019). A randomized trial of an online risk reduction intervention for young black MSM. AIDS Behav.

[57] Nelson KM, Perry NS, Stout CD, Dunsiger SI, Carey MP (2022). The young men and media study: a pilot randomized controlled trial of a community-informed, online HIV prevention intervention for 14-17-year-old sexual minority males. AIDS Behav.

[58] Visser M, Kotze M, van Rensburg MJ (2020). An mHealth HIV prevention programme for youth: lessons learned from the iloveLife.mobi programme in South Africa. AIDS Care.

[59] Periasamy M, Mohankumar V, Shanmugam V, Selvakumar M, Pandian SM, Sridharan L (2023). Redefining venereology practice in Tamil Nadu, South India—Nakshatra health—a networking model. Indian J Sex Transm Dis AIDS.

[60] Dulli L, Ridgeway K, Packer C, Murray KR, Mumuni T, Plourde KF, Chen M, Olumide A, Ojengbede O, McCarraher DR (2020). A social media-based support group for youth living with HIV in Nigeria (SMART Connections): randomized controlled trial. J Med Internet Res.

[61] Hill MJ, Coker S (2022). Novel use of video logs to deliver educational interventions to Black women for disease prevention. West J Emerg Med.

[62] Kurth AE, Sidle JE, Chhun N, Lizcano JA, Macharia SM, Garcia MM, Mwangi A, Keter A, Siika AM (2019). Computer-based counseling program (CARE+ Kenya) to promote prevention and HIV health for people living with HIV/AIDS: a randomized controlled trial. AIDS Educ Prev.

[63] Holst C, Stelzle D, Diep LM, Sukums F, Ngowi B, Noll J, Winkler AS (2022). Improving health knowledge through provision of free digital health education to rural communities in Iringa, Tanzania: nonrandomized intervention study. J Med Internet Res.

[64] Dike IC, Ebizie EN, Njoku OC, Oraelosi CA, Egbe CI, Nnamani AP, Ezeaku MN, Ihuoma EC, Otu MS, Okechukwu FO, Anowai CC, Nnodim EJ, Ukwuezeh CP, Onuorah AR, Onwuegbuchulam AC (2021). Improving knowledge and perception of HIV/AIDS among English language speaking children in rural areas through educational digital storytelling. Medicine (Baltimore).

[65] Tan RKJ, Koh WL, Le D, Banerjee S, Chio MT, Chan RKW, Wong CM, Tai BC, Wong ML, Cook AR, Chen MI, Wong CS (2022). Effect of a popular web drama video series on HIV and other sexually transmitted infection testing among gay, bisexual, and other men who have sex with men in Singapore: community-based, pragmatic, randomized controlled trial. J Med Internet Res.

[66] Tang W, Mao J, Liu C, Mollan K, Zhang Y, Tang S, Hudgens M, Ma W, Kang D, Wei C, Tucker JD (2019). Reimagining health communication: a noninferiority randomized controlled trial of crowdsourced intervention in China. Sex Transm Dis.

[67] Cho H, Porras T, Baik D, Beauchemin M, Schnall R (2018). Understanding the predisposing, enabling, and reinforcing factors influencing the use of a mobile-based HIV management app: a real-world usability evaluation. Int J Med Inform.

[68] Aomori M, Matsumoto C, Takebayashi S, Matsuyama N, Uto Y, Tanaka M, Samukawa S, Kato H, Nakajima H, Maeda H (2023). Effects of a smartphone app-based diet and physical activity program for men living with HIV who have dyslipidemia: a pilot randomized controlled trial. Jpn J Nurs Sci.

[69] Downing MJ, Wiatrek SE, Zahn RJ, Mansergh G, Olansky E, Gelaude D, Sullivan PS, Stephenson R, Siegler AJ, Bauermeister J, Horvath KJ, Chiasson MA, Yoon IS, Houang ST, Hernandez AJ, Hirshfield S (2023). Video selection and assessment for an app-based HIV prevention messaging intervention: formative research. Mhealth.

[70] Fisher JD, Fisher WA (1992). Changing AIDS-risk behavior. Psychol Bull.

[71] Bandura A, Freeman WH, Lightsey R (1999). Self-Efficacy: The Exercise of Control.

[72] Becker MH (1974). The health belief model and sick role behavior. Health Educ Monogr.

[73] Janz NK, Becker MH (1984). The health belief model: a decade later. Health Educ Q.

[74] Champion VL, Skinner CS (2008). The health belief model. Health Behav Health Educ Theory Res Pract.

[75] Ajzen I (1991). The theory of planned behavior. Organ Behav Hum Decis Process.

[76] Hevner AR (2007). A three cycle view of design science research. Scand J Inf Syst.

[77] Ogbuanya TC, Eseadi C, Orji CT, Anyanwu JI, Ede MO, Bakare J (2018). Effect of rational emotive behavior therapy on negative career thoughts of students in technical colleges in Nigeria. Psychol Rep.

[78] Ewart CK (1991). Social action theory for a public health psychology. Am Psychol.

[79] Bandura A, Walters RH (1977). Social Learning Theory.

[80] Zimmerman MA, Israel BA, Schulz A, Checkoway B (1992). Further explorations in empowerment theory: an empirical analysis of psychological empowerment. Am J Commun Psychol.

[81] Szapocznik J, Coatsworth JD (1999). An Ecodevelopmental Framework for Organizing the Influences on Drug Abuse: A Developmental Model of Risk and Protection.

[82] Ogden J (2016). Celebrating variability and a call to limit systematisation: the example of the behaviour change technique taxonomy and the behaviour change wheel. Health Psychol Rev.

[83] Gottlieb L, Rowat K (1987). The McGill model of nursing: a practice-derived model. ANS Adv Nurs Sci.

[84] Beck AT (1979). Cognitive Therapy and the Emotional Disorders.

[85] Miller S, Exner TM, Williams SP, Ehrhardt AA (2000). A gender-specific intervention for at-risk women in the USA. AIDS Care.

[86] Mayer RE (2005). The Cambridge Handbook of Multimedia Learning.

[87] Paivio A (1990). Mental Representations: A Dual Coding Approach.

[88] Petter S, DeLone W, McLean E (2017). Measuring information systems success: models, dimensions, measures, and interrelationships. Eur J Inf Syst.

[89] Jo P (2008). The transtheoretical model and stages of change. Health Behavior and Health Education: Theory, Research, and Practice.

[90] Mummah SA, Robinson TN, King AC, Gardner CD, Sutton S (2016). IDEAS (Integrate, Design, Assess, and Share): a framework and toolkit of strategies for the development of more effective digital interventions to change health behavior. J Med Internet Res.

[91] Ryan P (2009). Integrated theory of health behavior change: background and intervention development. Clin Nurse Spec.

[92] Fisher JD, Fisher WA (2000). Theoretical approaches to individual-level change in HIV risk behavior. Handbook of HIV Prevention.

