# Supplementary material for: Enhancing HIV Cognitive Abilities and Self-Management Through Information Technology–Assisted Interventions: Scoping Review
Source: J Med Internet Res. 2025 Jan 13;27:e57363. doi: 10.2196/57363 (PMC11773289; doi:10.2196/57363)
Supplement: Multimedia Appendix 1 [file jmir_v27i1e57363_app1.docx]

|  | Pubmed（n=61） |
| --- | --- |
| #1 | "HIV Infections"[Mesh] |
| #2 | ((((((((((((((HIV Patients[Title/Abstract]) OR (AIDS Patients[Title/Abstract])) OR (People Living HIV/AIDS[Title/Abstract])) OR (HIV-Positive Individuals[Title/Abstract])) OR (Individuals AIDS[Title/Abstract])) OR (HIV-Infected Persons[Title/Abstract])) OR (Persons Acquired Immunodeficiency Syndrome[Title/Abstract])) OR (HIV Seropositive Population[Title/Abstract])) OR (AIDS Survivors[Title/Abstract])) OR (HIV-Associated Patients[Title/Abstract])) OR (HIV/AIDS Affected Individuals[Title/Abstract])) OR (Patients HIV Disease[Title/Abstract])) OR (Individuals HIV Infections[Title/Abstract])) OR (AIDS-Related Patients[Title/Abstract])) OR (Seropositive AIDS Cases[Title/Abstract]) |
| #3 | #1 OR #2 |
| #4 | "Telemedicine"[Mesh] |
| #5 | ((((((((((((((((((((((((Electronic Health Information[Title/Abstract]) OR (eHealth[Title/Abstract])) OR (Digital Health[Title/Abstract])) OR (Health Information Technology[Title/Abstract])) OR (Mobile Applications[Title/Abstract])) OR (Mobile Health Apps[Title/Abstract])) OR (mHealth Apps[Title/Abstract])) OR (Health-related Apps[Title/Abstract])) OR (Online Health Education[Title/Abstract])) OR (Online Health Learning[Title/Abstract])) OR (Web-based Health Education[Title/Abstract])) OR (Internet Health Education[Title/Abstract])) OR (Information Technology Interventions[Title/Abstract])) OR (Information Technology Interventions[Title/Abstract])) OR (Technology-based Interventions[Title/Abstract])) OR (Computer-based Interventions[Title/Abstract])) OR (Health Promotion[Title/Abstract])) OR (Health Promotion Strategies[Title/Abstract])) OR (Health Communication[Title/Abstract])) OR (Health Information Promotion[Title/Abstract])) OR (social media intervention[Title/Abstract])) OR (Social networking technologies[Title/Abstract])) OR (social networks[Title/Abstract])) OR (social media technologies[Title/Abstract])) OR (social networking[Title/Abstract]) |
| #6 | #4 OR #5 |
| #7 | "Cognitive Training"[Mesh] |
| #8 | ((((((((((((((((((((((Cognitive Level[Title/Abstract]) OR (Cognitive Abilities[Title/Abstract])) OR (Cognitive Function[Title/Abstract])) OR (Cognitive Performance[Title/Abstract])) OR (Health Knowledge[Title/Abstract])) OR (Health Literacy[Title/Abstract])) OR (Health Understanding[Title/Abstract])) OR (Disease Knowledge[Title/Abstract])) OR (Self-management Skills[Title/Abstract])) OR (Self-care Skills[Title/Abstract])) OR (Health Self-efficacy[Title/Abstract])) OR (Disease Self-management[Title/Abstract])) OR (Health Literacy Skills[Title/Abstract])) OR (Health Information Literacy[Title/Abstract])) OR (Health Numeracy[Title/Abstract])) OR (Cognitive Improvement[Title/Abstract])) OR (Cognitive Enhancement[Title/Abstract])) OR (Cognitive Rehabilitation[Title/Abstract])) OR (Cognitive Training Effects[Title/Abstract])) OR (Patient Education Outcomes[Title/Abstract])) OR (Patient Knowledge Improvement[Title/Abstract])) OR (Patient Empowerment[Title/Abstract])) OR (Behavior Change[Title/Abstract]) |
| #9 | #7 OR #8 |
| #10 | #3 AND #6 AND #9 |

|  | Cochrane Library (n=361) |
| --- | --- |
| #1 | MeSH descriptor: [HIV Infections] explode all trees |
| #2 | (HIV Patients OR AIDS Patients OR People Living with HIV OR people living with AIDS OR HIV-Positive Individuals OR Individuals with AIDS OR HIV Infected Persons OR Persons Acquired Immunodeficiency Syndrome OR HIV Seropositive Population OR AIDS Survivors OR HIV Associated Patients OR HIV Affected Individuals OR AIDS Affected Individuals OR Patients with HIV Disease OR Individuals HIV Infections OR AIDS Related Patients OR Seropositive AIDS Cases):ti,ab,kw (Word variations have been searched) |
| #3 | #1 OR #2 |
| #4 | MeSH descriptor: [Telemedicine] explode all trees |
| #5 | (Electronic Health Information OR eHealth OR Digital Health OR Health Information Technology OR Mobile Applications OR Mobile Health Apps OR mHealth Apps OR Health-related Apps OR Online Health Education OR Online Health Learning OR Web-based Health Education OR Internet Health Education OR Information Technology Interventions OR Information Technology Interventions OR Technology-based Interventions OR Computer-based Interventions OR Health Promotion OR Health Promotion Strategies OR Health Communication OR Health Information Promotion OR social media intervention OR Social networking technologies OR social networks OR social media technologies OR social networking):ti,ab,kw |
| #6 | #4 OR #5 |
| #7 | MeSH descriptor: [Cognitive Training] explode all trees |
| #8 | (Cognitive Level OR Cognitive Abilities OR Cognitive Function OR Cognitive Performance OR Health Knowledge OR Health Literacy OR Health Understanding OR Disease Knowledge OR Self-management Skills OR Self-care Skills OR Health Self-efficacy OR Disease Self-management OR Health Literacy Skills OR Health Information Literacy OR Health Numeracy OR Cognitive Improvement OR Cognitive Enhancement OR Cognitive Rehabilitation OR Cognitive Training Effects OR Patient Education Outcomes OR Patient Knowledge Improvement OR Patient Empowerment OR Behavior Change) :ti,ab,kw |
| #9 | #7 OR #8 |
| #10 | #3 AND #6 AND #9 |

|  | Web of Science(n=44) |
| --- | --- |
| #1 | ((((((((((((((((TS=("HIV Patients")) OR TS=("AIDS Patients")) OR TS=("People Living with HIV")) OR TS=("people living with AIDS")) OR TS=("HIV-Positive Individuals")) OR TS=("Individuals with AIDS")) OR TS=("HIV Infected Persons")) OR TS=("Persons Acquired Immunodeficiency Syndrome")) OR TS=("HIV Seropositive Population")) OR TS=("AIDS Survivors")) OR TS=("HIV Associated Patients")) OR TS=("HIV Affected Individuals")) OR TS=("AIDS Affected Individuals")) OR TS=("Patients with HIV Disease")) OR TS=("Individuals HIV Infections")) OR TS=("AIDS Related Patients")) OR TS=("Seropositive AIDS Cases") and Preprint Citation Index |
| #2 | ((((((((((((((((((((((((((TS=("Telemedicine")) OR TS=("Electronic Health Information")) OR TS=("eHealth")) OR TS=("Digital Health")) OR TS=("Health Information Technology")) OR TS=("Mobile Applications")) OR TS=("Mobile Health Apps")) OR TS=("mHealth Apps")) OR TS=("Health-related Apps"))) OR TS=("Online Health Education")) OR TS=("Online Health Learning")) OR TS=("Web-based Health Education")) OR TS=("Internet Health Education")) OR TS=("Information Technology Interventions")) OR TS=("Information Technology Interventions")) OR TS=("Technology-based Interventions")) OR TS=("Computer-based Interventions")) OR TS=("Health Promotion")) OR TS=("Health Promotion Strategies")) OR TS=("Health Communication")) OR TS=("Health Information Promotion")) OR TS=("social media intervention")) OR TS=("Social networking technologies")) OR TS=("social networks")) OR TS=("social media technologies")) OR TS=("social networking") and Preprint Citation Index |
| #3 | (((((((((((((((((((((((TS=("Cognitive Training")) OR TS=("Cognitive Level")) OR TS=("Cognitive Abilities")) OR TS=("Cognitive Function")) OR TS=("Cognitive Performance")) OR TS=("Health Knowledge")) OR TS=("Health Literacy")) OR TS=("Health Understanding")) OR TS=("Disease Knowledge")) OR TS=("Self-management Skills")) OR TS=("Self-care Skills")) OR TS=("Health Self-efficacy")) OR TS=("Disease Self-management")) OR TS=("Health Literacy Skills")) OR TS=("Health Information Literacy")) OR TS=("Health Numeracy")) OR TS=("Cognitive Improvement")) OR TS=("Cognitive Enhancement")) OR TS=("Cognitive Rehabilitation")) OR TS=("Cognitive Training Effects")) OR TS=("Patient Education Outcomes")) OR TS=("Patient Knowledge Improvement")) OR TS=("Patient Empowerment")) OR TS=("Behavior Change") and Preprint Citation Index |
| #4 | #3 AND #2 AND #1 and Preprint Citation Index |

|  | CINAHL(n=6) |
| --- | --- |
| #1 | SU HIV Infections OR SU ( hiv patients or aids patients ) |
| #2 | SU ( telemedicine or telehealth or ehealth or e-health or mhealth or m-health ) OR SU electronic health information OR SU Information Technology Interventions OR SU technology-based intervention OR SU social media intervention OR SU Social networking technologies |
| #3 | SU ( cognitive training or brain training or executive function training ) OR SU ( cognitive abilities or cognitive function ) OR SU ( health knowledge or awareness or understanding ) OR SU cognitive improvement OR SU Cognitive Training Effects OR SU Patient Knowledge Improvement |
| #4 | S1 AND S2 AND S3 |

|  | EMBASE(n=254) |
| --- | --- |
| #1 | 'human immunodeficiency virus infection'/exp OR 'human immunodeficiency virus infection' |
| #2 | 'hiv patients':ab,ti OR 'aids patients':ab,ti OR 'human immunodeficiency virus infected patient':ab,ti OR 'people living with aids':ab,ti OR 'hiv-positive individuals':ab,ti OR 'individuals with aids':ab,ti OR 'hiv infected persons':ab,ti OR 'persons acquired immunodeficiency syndrome':ab,ti OR 'hiv seropositive population':ab,ti OR 'aids survivors':ab,ti OR 'hiv associated patients':ab,ti OR 'hiv affected individuals':ab,ti OR 'aids affected individuals':ab,ti OR 'patients with hiv disease':ab,ti OR 'individuals hiv infections':ab,ti OR 'aids related patients':ab,ti OR 'seropositive aids cases':ab,ti |
| #3 | #1 OR #2 |
| #4 | 'telemedicine'/exp OR 'telemedicine' |
| #5 | 'electronic health information':ab,ti OR 'ehealth':ab,ti OR 'digital health':ab,ti OR 'digital health technology':ab,ti OR 'digital health intervention':ab,ti OR 'medical informatics':ab,ti OR 'mobile applications':ab,ti OR 'mobile health apps':ab,ti OR 'mhealth apps':ab,ti OR 'health-related apps':ab,ti OR 'online health education':ab,ti OR 'online health learning':ab,ti OR 'web-based health education':ab,ti OR 'internet health education':ab,ti OR 'information technology interventions':ab,ti OR 'technology-based interventions':ab,ti OR 'computer-based interventions':ab,ti OR 'health promotion':ab,ti OR 'health promotion strategies':ab,ti OR 'medical information':ab,ti OR 'health information promotion':ab,ti OR 'social media intervention':ab,ti OR 'social networking technologies':ab,ti OR 'social networks':ab,ti OR 'social media technologies':ab,ti OR 'social network':ab,ti |
| #6 | #4 OR #5 |
| #7 | 'cognition'/exp |
| #8 | 'cognitive training':ab,ti OR 'cognitive level':ab,ti OR 'cognitive abilities':ab,ti OR cognition:ab,ti OR 'mental performance':ab,ti OR 'attitude to health':ab,ti OR 'health literacy':ab,ti OR 'health understanding':ab,ti OR 'disease knowledge':ab,ti OR 'self-management skills':ab,ti OR 'self-care skills':ab,ti OR 'health self-efficacy':ab,ti OR 'disease self-management':ab,ti OR 'health literacy skills':ab,ti OR 'health information literacy':ab,ti OR 'health numeracy':ab,ti OR 'cognitive improvement':ab,ti OR 'cognitive enhancement':ab,ti OR 'cognitive rehabilitation':ab,ti OR 'cognitive training effects':ab,ti OR 'patient education outcomes':ab,ti OR 'patient knowledge improvement':ab,ti OR 'patient empowerment':ab,ti OR 'behavior change':ab,ti |
| #9 | #7 OR #8 |
| #10 | #3 AND #6 AND #9 AND [2017-2023]/py |
